# First Report of Human Urinary Tract Infection Caused by *Lactococcus petauri*

**DOI:** 10.3390/microorganisms11102583

**Published:** 2023-10-18

**Authors:** Silvia Colussi, Paolo Pastorino, Marino Prearo, Simona Sciuto, Fabio Bondavalli, Pier Luigi Acutis, Elena Bozzetta, Francesco Amisano, Angelo Salerno

**Affiliations:** 1 Istituto Zooprofilattico Sperimentale del Piemonte, Liguria e Valle d’Aosta, 10154 Turin, Italy; silvia.colussi@izsto.it (S.C.); marino.prearo@izsto.it (M.P.); simona.sciuto@izsto.it (S.S.); fabio.bondavalli@izsto.it (F.B.); pierluigi.acutis@izsto.it (P.L.A.); elena.bozzetta@izsto.it (E.B.); 2 Scuola di Specializzazione in Microbiologia e Virologia, Dipartimento di Scienze Clinico-Chirurgiche, Diagnostiche e Pediatriche, Università Degli Studi di Pavia, 27100 Pavia, Italy; francesco.amisano01@universitadipavia.it; 3 S.S. Microbiologia, Ospedale SS Antonio e Margherita, 15057 Tortona, Italy; asalerno@aslal.it

**Keywords:** *Lactococcus garvieae*, urinary tract infection, lactococcosis

## Abstract

*Lactococcus petauri* is a recently described species of the genus *Lactococcus*. It was reported as an etiological agent of piscine lactococcosis together with *Lactococcus garvieae*. *L. garvieae* was already described as an opportunistic pathogen in human infections, with a potential zoonotic role. This paper represents the first report of a human urinary tract infection caused by *L. petauri*. A 91-year-old man was admitted to the emergency department for a femur fracture consequent to a domestic accident. The fracture was reduced by surgery and a catheterized specimen urine culture revealed a high bacterial load sustained by Gram-positive cocci, identified by Vitek 2 compact as *L. garvieae*, and subsequently as *L. petauri* through Internal Transcribed spacer 16S-23S r-RNA amplification. The number of *L. petauri* infections in humans is expected to rise in the near future mainly due to diagnostic improvement. A dedicated survey on *L. garvieae* and *L. petauri* infections in humans should be performed to better understand their role as pathogens and as zoonotic agents.

## 1. Introduction

*Lactococcus garvieae* is a Gram-positive coccus responsible for lactococcosis, an infectious disease well known in aquaculture for its high economic impact on marine and fresh-water production. Lactococcosis is a septicaemic infection in fish, that show clinical signs such as lethargy, anorexia, hypermelanosis, exophthalmos, spleen enlargement, enteritis, and diffuse haemorrhage [1]. Multi-drug resistance represents a big issue associated with this pathogen in aquaculture [2]. The host range is not limited to aquatic species. This agent has also been found in cows with subclinical intramammary infections, porcine blood, cat and dog tonsils, raw cow’s milk, and meat products [1]. Moreover, *L. garvieae* has been described as an opportunistic pathogen for humans, mainly affecting immunosuppressed patients, presenting co-morbidity factors such as age and concomitant diseases, though different cases have been reported in young and immunocompetent patients too [3,4]. It has mainly been isolated in cases of endocarditis [5,6], bacteriemia [3,7], peritonitis [3], liver abscess [8], osteomyelitis [9], spondylodiscitis [10], meningitis [11] and urinary tract infections [12,13]. Recently, a case of paediatric sepsis and the first description of recurrent throat infection in two children have been reported, both associated with multi-drug resistance [4,14]. The increasing number of human infections in the recent years has raised awareness of the importance of *L. garvieae* as an emerging zoonotic pathogen associated with the handling or ingestion of raw fish and seafood [3,10] and cheese [15,16]. *L. garvieae* has been considered as the only etiological agent of piscine lactococcosis, until recently, when genomic analysis reassigned some *L. garvieae* strains isolated in fish to the new species, *L. petauri*, described in 2017 by Goodman in the sugar glider (*Petaurus breviceps*) [17]. Until now, *L. petauri* has been reported as the causative agent of natural outbreaks in rainbow trout (*Oncorhynchus mykiss*) [18,19] and in Nile tilapia (*Oreochromis niloticus*) [20]. Lactococcosis is traditionally diagnosed in the laboratory by conventional microbiological techniques or commercial phenotypic and biochemical tests. Both *L. garvieae* and *L. petauri* have similar phenotypic and biochemical properties [17] and for this reason the most widely used commercial phenotypic methods for a routine identification based on Analytical Profile Index (API) and matrix-assisted laser desorption ionization time-of-flight mass spectrometry (MALDI-TOF MS) did not distinguish between these two species [20].

Here, a case of urinary tract infection caused by *L. petauri* in a man is described for the first time.

## 2. Materials and Methods

### 2.1. Case Description

A 91-year-old man was admitted to the emergency department for a suspected sub-capital femur fracture subsequent to a fall in a domestic accident. Despite a negative radiograph, the computed tomography imaging confirmed sub-capital femur fracture and the patient was transferred to the traumatology division. The patient had an history of prostate cancer treated with hormone therapy, type II diabetes mellitus and atrial fibrillation treated with Edoxaban therapy. Moreover, upon further interview, the patient reported long-term pantoprazole use.

Physical examination at admission showed that the patient had a blood pressure of 99/60, arterial oxygen saturation measured by pulse oximetry (SpO_2_) 98%, normal temperature, irregular cardiac arrhythmia sound and the electrocardiogram EKG cardiology evaluation confirmed a history of atrial fibrillation of the heart. Laboratory data on admission showed red blood cells (RBC) and platelet counts in the normal range (4.92 × 10^6^/µL and 153 × 10^3^/µL); haemoglobin 15.3 g/dL) and normal leucocyte count (white blood cells 4.41 × 10^6^/µL); C-reactive protein (CRP 1.82 mg/dL; normal value < 0.5 mg/dL); lactate dehydrogenase (LDH 394 UI/L; normal value 40–480 UI/L); rheumatic factor within normal range. The creatinine was 0.96 mg/dL, level blood glucose 139 mg/dL and prostate-specific antigen (PSA) levels of 5.64 ng/mL. Urinalysis showed haematuria and leucocytes eight days post-catheterization.

The fracture was reduced by hip arthroplasty surgery and anti-thrombo-embolic therapy with low-molecular-weight heparin (LMWH) and antibiotic prophylaxis with Cefazolin 2 gr. iv. to pre-anesthesia in a single administration (very-short-shot) which were administered. A radiographic evaluation was performed 6 weeks after the surgical procedure. The consolidation of peri-prosthetic fractures occurred at about 3 months post-surgery.

From the analysis of the patient’s eating habits, it emerged that he was a frequent consumer of milk and soft cheeses.

### 2.2. Microorganism Identification

Catheterized specimen urine culture was carried out using semiautomated streaking. A ball-based bench-top WASP (Copan, Italy) was used with 10 μL inocula of suspension to the following solid media: Columbia Blood Agar, and MacConkey Agar (BioMérieux, Marcy l’Etoile, France). A pure culture of Gram-positive, catalase-negative cocci was isolated on Columbia blood agar with the production of α-haemolysis. Microbial identification was performed using a Vitek2^®^ Compact automatic system using a GP VITEK2-card (BioMérieux, Marcy l’Etoile, France).

### 2.3. Molecular Identification

The boiling and freeze-thawing method was used for DNA extraction and the 16S–23S rRNA Internal Transcribed Spacer (ITS) region was amplified by PCR using the primers 16S 5′-GCTGGATCACCTCCTTTCT-3′ and 23S 5′-GGTACTTAGATGTTTCAGTTCC-3′ as described by Stoppani et al. [21]. Amplicons were purified with Qiaquick purification kit (Qiagen) and bi-directionally sequenced using the Brilliant Dye Terminator (v1.1) Cycle Sequencing Kit (Nimagen, BV, Nijmegen, Netherlands) on the genetic analyser (Applied Biosystems 3130XL, Applied Biosystems, Carlsbad, USA). DNA sequence analysis was performed with DNASTAR Lasergene Software v. 5.0.1 (DNASTAR, Madison, USA). Consensus sequence obtained from the alignment of the forward and reverse sequences was compared with nucleotide sequences in the GenBank database using the Basic Local Alignment Search Tool (BLAST) algorithm [22].

### 2.4. Heamolysins Presence Evaluation

Haemolysins were evaluated as pathogenicity factors. The three hemolysin genes (*Hly1*, *Hly2* and *Hly3*) were amplified starting from genomic DNA, extracted as reported in Section 2.2, following the protocol previously described by Teker et al. [23]. Amplicons were run on a Gelgreen (Biotium, Fremont, CA, USA) stained agarose gel and then visualized under UV exposure. A 50–2000 kb ladder (Amplisize Molecular Ruler, Bio-Rad, Hercules, CA, USA) was used as a molecular marker.

### 2.5. Antimicrobial Resistance Evaluation

Concerning antibiotic susceptibility testing, Vitek^®^ 2 is not the gold standard for *Lactococcus* spp. For this reason, E-test antibiotic susceptibility testing was used because it is described as the most reliable [24]. The following antimicrobial agents were tested: penicillin, clindamycin, tetracycline, trimethoprim/sulfamethoxazole, ceftriaxone, meropenem, erythromycin and levofloxacin.

## 3. Results

### 3.1. Laboratory Data Analysis

Laboratory data submitted by the emergency department showed: red blood cells (RBC) and platelet counts in the normal range (4.92 × 10^6^/µL and 153 × 10^3^/µL); haemoglobin (15.3 g/dL), and a normal leucocyte count (white blood cells 4.41 × 10^6^/µL). C-reactive protein (CRP 1.82 mg/dL; normal value < 0.5 mg/dL), lactate dehydrogenase (LDH 394 UI/L; normal value 40–480 UI/L), rheumatic factor within normal range; the pre-therapeutic creatinine was 0.96 mg/dL.

At eight days post-surgery, urinalysis and urine culture were performed due to the persistent need to urinate and suprapubic pain. The microscopic examination of urine sediments showed the presence of haematuria and leucocytes. Moreover, the Gram-positive cocci bacterial load was 10^6^ CFU/mL. Erythrocyte sedimentation was significantly higher (72 mm/h vs. <10 mm/h).

### 3.2. Microorganism Identification

The automated system, Vitek^®^ Compact, correctly identified *Lactococcus garvieae*, as an excellent identification (98%), without requiring additional tests. A specifically developed molecular method discriminating between *L. garvieae* and *L. petauri*, based on ITS, was used. This method revealed the presence of *L. petauri* with a percentage of similarity of BLAST of 99.41% with the reference sequences reported for all the *L. petauri* strains isolated from rainbow trout in Spain, Turkey, and Greece (GenBank accession numbers OQ108346, OQ108345, OQ108344). Nevertheless, this human strain presented one deletion (g.42_43 delA) and two SNPs at position g.172G > A and g.173A > C (numbering refers to the reference sequence reported above). The human *L. petauri* sequence was deposited on Genebank (accession number OR231105).

### 3.3. Haemolysins

The strain was positive for haemolysin 2 and negative for haemolysin 1 and 3.

### 3.4. Antimicrobial Resistance

According to the interpretative criteria and testing method of the Clinical and Laboratory Standards Institute (CLSI M45 3rd edition) [25], cation-adjusted Mueller–Hinton broth supplemented with lysed horse blood (CAMHB-LHB) and antimicrobial susceptibility breakpoints test-method was used to determine the minimum inhibitory concentration (MIC), using Sensititre (Trek Diagnostic Systems, Thermo Fisher Scientific, Cleveland, OH, USA). ATCC (American Type Culture Collection) *S. pneumoniae* 49619 was used. Susceptibility breakpoint measurements for *Lactococcus* spp., including penicillin and ampicillin (MIC ≤ 1), ceftriaxone (MIC ≤ 1), meropenem (MIC ≤ 0.25), tetracycline (MIC ≤ 2) erythromycin (MIC ≤ 0.5), clindamycin (MIC ≤ 0.5) and vancomycin (MIC ≤ 2) were carried out and our strain was considered resistant to clindamycin, tetracycline, trimethoprim/sulfamethoxazole and susceptible to penicillin, ceftriaxone, meropenem, erythromycin and levofloxacin. *Lactococcus garvieae* is intrinsically resistant to clindamycin and resistant to tetracycline due to the presence of *tetM* or *tetS*. Specific indications for *L. petauri* are not reported. MIC values are reported in Table 1.

## 4. Discussion and Conclusions

*Lactococcus petauri* has so far been reported in fish such as trout and Nile tilapia and in cheese; in particular, strain INF110 has been isolated from traditional Montenegrin brine cheese [16]. This pathogen is widely spread in the Mediterranean area [21]; however, the real prevalence and incidence are still unknown, mainly due to the diagnostic misidentification of the species, and there is a lack of data to allow the evaluation of its zoonotic role.

The present study describes the first case of human urinary tract infection caused by *L. petauri*. This is also the first case reported in Italy where, at the moment of writing, no cases have been reported in fish. Data from archival strains collected in Italy, Spain, Greece, and Turkey showed the predominant presence of *L. petauri* in the Mediterranean area except for Italy where only *L. garvieae* species was detected by Stoppani et al. [21]. The very similar pathogen *L. garvieae* likely causes opportunistic infections in body systems with co-morbidity factors, as reported here for old age, history of prostate cancer, type II diabetes mellitus and atrial fibrillation.

The most probable route of diffusion is the bloodstream, entering from diseased sites in the gastrointestinal tract after the ingestion of contaminated food. Favoured by the concomitant use of gastroprotective drugs, this may allow the pathogen to pass the gastric acid barrier. In this case-report study, the patient described was a long-term pantoprazole user and raw milk and cheese would have been the most probable route of contact of *L. petauri*. Post-surgery antibiotic treatment based on cefazoline, used as prophylaxis, was not sufficient to eradicate the pathogen from the urinary tract. In this case, in fact, extended periods and higher dosages are required. Despite this, the infection was related to the catheterization and therefore resolved with the removal of the catheter, as required by the protocol of uncomplicated urinary tract infections. Monthly microbiological tests did not show the persistence of *L. petauri* infection.

Interestingly the ITS region amplified for diagnostic purposes revealed different single nucleotide polymorphisms compared to the ones reported in strains isolated from trout in different countries [21], even if maintaining the single nucleotide polymorphisms (SNPs) described as diagnostic for species discrimination. The role of the three haemolysins as virulence factors is still controversial. Teker et al. [23] found *hly*1 and 2 as key virulence factors in *L. garvieae* when compared with *hly* 3. In this strain isolated from a human, only *hly* 2 was present; this result was in line with what was found in fish where *hly* 3 was always absent and *hly* 1 amplified with a very low percentage [26]. This could indicate a different role of haemolysins in *L. petauri* compared to *L. garvieae*, but data are still lacking. The number of *L. petauri* infections in humans is expected to rise in the near future, mainly due to the improvement of diagnostic tools not requiring genome analysis. A dedicated survey on *L. garvieae* and *L. petauri* infections in humans, supported by epidemiological studies to trace back infection related to food should be carried out, as well as a survey on antimicrobial resistance associated with plasmid analysis to understand the main route of transmission of antimicrobial resistance genes in this pathogen.

## Figures and Tables

**Table 1:** Pattern of antibiotic susceptibility of *L. petauri* isolated from a human.

Antibiotic	MIC (μg/mL)	Interpretation *
Penicillin	0.5	S
Ampicillin	0.5	S
Ceftriaxone	0.25	S
Meropenem	0.032	S
Tetracycline	256	R
Levofloxacin	1	S
Trimethoprim-sulfamethoxazole	>32	R
Clindamycin	128	R

* Interpretative criteria of CLS M45 3rd edition [25]. S = sensitive; R = resistant.

## Data Availability

ITS sequence for strain 1LP_H was deposited on 11 July 2023 to GenBank (www.ncbi.nlm.nih.gov/genbank; accessed on 11 July 2023) under the following accession number: OR231105.
